# Primary pulmonary NUT carcinoma: pathological features, clinical diagnosis, treatment, and prognosis — An extended review

**DOI:** 10.3389/or.2026.1967215

**Published:** 2026-09-28

**Authors:** Shan Wu, Miao Zhang, Hui Wang

**Affiliations:** Department of Oncology, Tianjin Union Medical Center, The First Affiliated Hospital of Nankai University, Nankai University, Tianjin, China

**Keywords:** diagnosis, NUT carcinoma, NUTM1 gene rearrangement, primary pulmonary neoplasm, prognosis, treatment

## Abstract

NUT carcinoma is a rare and aggressive lung tumor driven by NUTM1 gene rearrangements. It accounts for less than 1% of all lung cancers, with fewer than 100 cases reported worldwide. The disease is often misdiagnosed due to non-specific symptoms and histological overlap with other tumors. This review provides an updated overview of its pathological features, clinical presentation, diagnosis, treatment, and prognosis, aiming to serve as a practical reference for clinicians and pathologists.

## 1 Introduction

NUT carcinoma was first described in 1991 through two independent case reports, both characterized by a t (15; 19) chromosomal translocation (1, 2). In 2003, French et al. revealed that this translocation generates a fusion oncogene between the BRD4 gene and the NUT gene (3). Since the tumor was originally thought to arise exclusively in midline strictures (such as the nasal cavity, palate, and mediastinum), it was termed “NUT midline carcinoma.” However, subsequent case reports have documented NC occurring in non-midline organs, with the lung being one of the most common non-midline sites. In 2015, the World Health Organization (WHO) formally classified primary pulmonary NUT carcinoma under “other undifferentiated carcinomas” of the lung (4). In the 5th edition of the WHO Classification of Thoracic Tumours (2021), NUT carcinoma, together with thoracic SMARCA4-deficient undifferentiated tumour, was grouped under “molecularly defined thoracic tumours” (5).

With the widespread application of NUT-specific antibodies and the increasing availability of molecular testing, the recognition of NUT carcinoma is gradually improving. Nevertheless, because the disease has long been under-recognized, clinicians often lack awareness, leading to many missed or misdiagnosed cases. Li et al. reported that primary pulmonary NUT carcinoma accounts for approximately 0.22% of all primary thoracic tumors (6). The average survival of pulmonary NUT carcinoma is only about 6–7 months, making it one of the lung cancer subtypes with the poorest prognosis.

Molecularly defined tumours represent a significant recent advance in thoracic pathology. In a comprehensive review, Roden pointed out that molecular studies now allow us to better characterise these tumours and understand their pathogenesis (7). In the thorax, molecularly defined tumours include NUT carcinoma, thoracic SMARCA4-deficient undifferentiated tumour, primary pulmonary myxoid sarcoma, and others. Although rare, with the increasing use of immunostains such as NUT, BRG1, INI-1, and cytogenetic studies like EWSR1 rearrangements, pathologists are now able to identify and diagnose these tumours more accurately than before.

Recently, Luo et al. proposed that NUT carcinoma should be recognized as an unrecognized subtype of squamous cell carcinoma of the lung and head/neck, based on transcriptomic, pathological, and cellular-of-origin data (8). This reclassification, if adopted, could significantly improve clinical awareness and accelerate the development of effective treatments for this aggressive malignancy. The recently published international guidelines on the diagnosis and treatment of NUT carcinoma provide the first consensus recommendations on diagnostic algorithms, BET inhibitor use, and multidisciplinary management. These guidelines recommend NUT IHC as an initial screening for malignant tumors exhibiting a poorly differentiated phenotype (9) (Table 1).

**TABLE 1:** Expression patterns of other IHC markers

Marker	Positivity rate	Remarks
p63	∼87%	More sensitive than p40
p40	∼65%	More specific but less sensitive
Pan-cytokeratin	∼74%	Epithelial marker; occasional entirely negative cases
CK5/6	Often positive	—

## 2 Pathological features

### 2.1 Histological features

Primary pulmonary NUT carcinoma typically exhibits an undifferentiated or poorly differentiated morphology. The classic microscopic appearance consists of monotonous small-to-medium-sized round cells with scant eosinophilic cytoplasm, arranged in solid sheets, nests, or cords (10, 11).

Abrupt keratinization is the most characteristic histological finding of NUT carcinoma—the sudden appearance of single cells or small clusters of squamous differentiation against a background of undifferentiated tumor cells (10, 12). This feature helps distinguish NUT carcinoma from other undifferentiated tumors. In addition, the tumor often shows the following accompanying features (13–16):

- Extensive necrosis: widespread tumor necrosis is common.
- Neutrophilic infiltration: abundant neutrophils are relatively characteristic.
- Active mitotic figures: reflecting the high proliferative activity.
- Apoptotic bodies: observed in some tumor cells.

In an analysis of 7 cases of primary pulmonary NUT carcinoma, Zhuang et al. found that 4 cases showed areas of abrupt keratinization, mitoses were easily identified, and extensive necrosis was present (17). A more recent study of 21 pulmonary NUT carcinomas further delineated the morphological spectrum—ranging from the classic “small round blue cell” pattern with abrupt keratinization to variants composed predominantly of primitive undifferentiated cells (18).

### 2.2 Immunohistochemical features

Diffuse nuclear positivity for NUT protein is the gold standard for confirming the diagnosis of NUT carcinoma. Using the rabbit monoclonal antibody C52B1, positivity is defined as speckled nuclear staining in >50% of tumor cells (19). This technique has a diagnostic sensitivity of 87%, a specificity approaching 100%, a negative predictive value of 99%, and a positive predictive value of 100% (13, 19, 20).

The expression patterns of other common immunohistochemical markers are summarized below.

## 3 Epidemiology and demographics

### 3.1 Age distribution

Primary pulmonary NUT carcinoma predominantly affects young to middle-aged individuals. In a comprehensive review by Yuan et al. including 55 patients, the mean age was 41.4 years (range 6–82) and the median age was 36 years (14). The age distribution was: 0–30 years (17 cases), 31–60 years (26 cases), and >60 years (12 cases).

A systematic review by Zhao et al. (including 62 cases) reported a median age at diagnosis of 29.5 years (21). Another study noted that pulmonary NUT carcinoma typically occurs in non-smokers under 40 years of age (22). Unlike classic NUT carcinoma, which preferentially affects adolescents and young adults (median age ∼22 years), primary pulmonary NUT carcinoma shows a broader age range, and older patients are not exempt (12). Older age at diagnosis is associated with lower cumulative survival (23).

### 3.2 Sex distribution

Primary pulmonary NUT carcinoma is slightly more common in males. In the study by Yuan et al., there were 36 males and 19 females, with a male-to-female ratio of approximately 1.89:1 (14). However, other sources indicate that the incidence between sexes is roughly equal (24). In Zhao et al.'s systematic review, male sex was identified as one of the indicators of shorter survival (21).

### 3.3 Smoking history

The disease predominantly occurs in non-smokers. Among the 42 patients with documented smoking history in Yuan et al.'s review, 28 (66.7%) were non-smokers and 14 (33.3%) were smokers, yielding a ratio of 2:1 (14). This contrasts sharply with other lung malignancies such as squamous cell carcinoma, which are strongly associated with smoking, and thus carries important differential diagnostic significance. This observation aligns with the proposal by Luo et al. that NUT carcinoma represents a distinct subtype of squamous cell carcinoma with a unique biology (8).

## 4 Clinical symptoms

Primary pulmonary NUT carcinoma lacks specific clinical manifestations, which is a major reason for its high misdiagnosis rate.

In the analysis by Yuan et al. of 44 patients with detailed symptom records, the results were as follows (14) (Table 2):

**TABLE 2:** Clinical symptoms records in NUT patients.

Symptom	Frequency
Cough	63.6% (28/44)
Dyspnea	29.5% (13/44)
Chest pain	18.2% (8/44)
Hemoptysis	18.2% (8/44)
Wheezing	15.9% (7/44)

Zhao et al.'s systematic review reported that the most common presenting symptoms were cough (47.50%) and chest/back pain (37.50%) (21). Other symptoms included fever, chest tightness, sputum production, weight loss, fatigue, neck discomfort, anorexia, night sweats, and hoarseness. Most patients are already at an advanced stage at the time of presentation. The median interval from symptom onset to diagnosis is 10 weeks (range 1–165 weeks), and diagnostic delay is common (25). Atypical presentations are also noteworthy. Several case reports described primary pulmonary NUT carcinoma with paraneoplastic hypercalcemia (26, 27), highlighting that clinicians should consider NUT carcinoma even in elderly patients, and that atypical features such as hypercalcemia may facilitate early detection.

## 5 Clinical diagnosis

### 5.1 Imaging features

Primary pulmonary NUT carcinoma typically appears on CT as a large, irregular, lobulated soft-tissue mass. The main imaging features include (21, 22, 28–30):

- Size: maximum diameter 1.4–12.7 cm, mean 6.55 cm; 85% of lesions >3 cm
- Predominant location: lower lobe in 46.5%, with both lungs affected (right slightly more frequent)
- Morphology: lobulated growth, with homogeneous or heterogeneous density
- Enhancement: mild enhancement on contrast-enhanced CT
- Lymph node metastasis: extremely common (91.9%), most frequently mediastinal (79.3%) and hilar (48.3%)
- Distant metastasis: about 96% of pulmonary NUT carcinomas have distant metastases; the most common sites are bone (71.4%), liver (23.8%), and adrenal glands (14.3%)
- Other findings: pleural effusion (associated with shorter survival), compressive atelectasis, or necrotic foci in some cases.
- PET/CT typically shows increased ^18^F-FDG uptake in the primary lesion and metastatic areas (31). A unilateral large pulmonary mass is a common presentation, sometimes accompanied by pleural effusion or atelectasis.

### 5.2 Diagnostic challenges and misdiagnosis

The diagnosis of primary pulmonary NUT carcinoma is highly challenging for several reasons (16, 20, 32–35):

- Morphological overlap: significant overlap with other poorly differentiated tumors (poorly differentiated squamous cell carcinoma, small cell carcinoma, thoracic SMARCA4-deficient undifferentiated tumour, etc.).
- Misleading immunophenotype: expression patterns of p63/p40 and TTF-1 can mislead the diagnosis.
- Small biopsy specimens: particularly prone to misdiagnosis.
- Insufficient clinical awareness: due to rarity, clinicians lack vigilance.

Common misdiagnoses include: non-small cell lung carcinoma (especially squamous cell carcinoma), small cell lung carcinoma, neuroendocrine tumors, lymphoma, sarcoma, Ewing sarcoma, thymic carcinoma (basaloid variant) etc. NUT carcinoma and thoracic SMARCA4-deficient undifferentiated tumor are distinct entities in the 5th edition of the WHO classification; both require prompt testing on biopsy specimens, but the broad differential diagnosis poses a challenge to pathologists.

### 5.3 Diagnostic workup

A definitive diagnosis relies on NUT immunohistochemistry or molecular testing. Recommended diagnostic algorithm:

- Screening: For undifferentiated or poorly differentiated thoracic carcinomas, especially in young non-smoking patients, routine NUT immunohistochemical screening should be performed.
- Confirmation: NUT protein immunohistochemistry (C52B1 monoclonal antibody); diffuse nuclear positivity in >50% of tumor cells confirms the diagnosis.
- Verification: FISH to detect NUTM1 gene break-apart rearrangement.
- Supplementary: RT-PCR or NGS to identify the specific fusion partner.

Important note: If NUT immunostaining is negative, the diagnosis of NC should not be entirely excluded; molecular analysis by FISH or NGS should be performed to test for NUTM1 rearrangement. The final diagnosis of NUT carcinoma requires confirmation of the presence of NUTM1 gene rearrangement. A recent multi-omics study by Kim et al. on 116 NUT carcinoma patients found that standard DNA-based NGS detected only 21.6% (22/102) of cases, while RNA-based fusion detection achieved 83.9% (52/62) and NUT IHC achieved 100% (99/99) (36). These findings have direct implications for the diagnostic workup, emphasizing the critical role of RNA-based testing and IHC in detecting NUTM1 rearrangements.

### 5.4 Key differential diagnostic points

- vs. poorly differentiated squamous cell carcinoma: NUT carcinoma often shows p63-positive but p40-negative or weakly positive, whereas squamous carcinoma is typically positive for both (32, 37).
- vs. thoracic SMARCA4-deficient undifferentiated tumour: the latter shows loss of SMARCA4 (BRG1) expression, whereas NUT carcinoma retains SMARCA4 (7).
- vs. small cell lung carcinoma: NUT carcinoma is usually negative or only focally positive for neuroendocrine markers (34).
- vs. lymphoma: NUT carcinoma is cytokeratin-positive and LCA-negative (32).

## 6 Treatment

There is currently no standardized treatment protocol for primary pulmonary NUT carcinoma. The recently published international guidelines on the diagnosis and treatment of NUT carcinoma provide the first consensus recommendations on diagnostic algorithms, BET inhibitor use, and multidisciplinary management. Existing therapeutic modalities include surgery, chemotherapy, radiotherapy, immunotherapy, and targeted therapy. Due to the lack of randomized controlled trials, treatment recommendations are largely based on case reports and retrospective studies.

### 6.1 Surgery

For early-stage, resectable cases, surgical resection is the preferred treatment. In the review by Yuan et al:

- Patients receiving surgery alone (n = 7) had a mean OS of 3.54 months.
- Those receiving neoadjuvant therapy or postoperative adjuvant chemoradiotherapy (n = 4): 3 were still alive at the time of reporting, and 1 had an OS of 8 months.
- Surgical resection combined with adjuvant therapy yields better outcomes.

However, because most tumors are centrally located, adjacent to the hilum and mediastinum, and because most patients are already at an advanced stage at diagnosis, surgical accessibility is limited. In the case series published in BMC Cancer, most pulmonary NUT carcinomas were centrally located (38).

### 6.2 Chemotherapy

Chemotherapy is the most commonly used systemic therapy for pulmonary NUT carcinoma. Platinum-based combination regimens are most frequently used (14, 39–44):

- TP regimen (taxane + platinum): most common (30.3%)
- EP regimen (etoposide + platinum): 18.2%
- Others: DP (docetaxel + platinum), AP (pemetrexed + platinum), VAI regimen, etc.

Patients receiving chemotherapy had a mean OS of 7.32 months, better than the untreated group (1.3 months) and the surgery-alone group (3.54 months). For patients receiving chemotherapy alone, the mean OS was 5.58 months. In the German cohort of 35 adult NC patients, 80% received multi-agent combination chemotherapy (39). However, conventional single-agent or standard multimodal therapy (chemotherapy ± radiotherapy) has limited efficacy, with a median PFS of only 2.1 months (40). Early sensitivity followed by rapid resistance is an important feature of chemotherapy. One case report showed that first-line and second-line treatments were effective for the first two cycles, extending OS to >8 months, but rapid resistance subsequently developed (41). Another study noted that pulmonary NUT carcinoma shows only transient responses to chemotherapeutic agents, underscoring the need for more effective strategies (42–44).

### 6.3 Radiotherapy

Pulmonary NUT carcinoma is sensitive to radiotherapy, which is an important therapeutic feature. Concurrent or sequential chemoradiotherapy is currently the most effective treatment strategy for prolonging overall survival.

The 6 patients receiving chemoradiotherapy had a mean OS of 13.2 months, with 4 surviving >12 months (12.9, 16, 18, and 26.7 months, respectively). This was significantly better than chemotherapy alone (5.58 months) and surgery alone (3.54 months) (14).

Advances in radiotherapy techniques offer additional options. One case report described dose-escalated adaptive radiotherapy for primary pulmonary NUT carcinoma with NSD3-NUTM1 fusion, demonstrating feasibility in patients with disseminated disease and impaired pulmonary function. Hypofractionated radiotherapy (37.5 Gy/15 fractions) can control primary/mediastinal lesions (45).

Multimodal therapy combining anti-angiogenic agents with platinum-based chemotherapy and radiotherapy also shows promise. Two patients receiving this combination achieved OS of 32 months and 13 months, far exceeding the currently reported median survival for advanced NUT carcinoma (46).

### 6.4 Immunotherapy

The efficacy of immunotherapy in pulmonary NUT carcinoma remains uncertain, with overall limited benefit. The main reasons include (39):

- Low PD-L1 expression in general.
- Immunologically “cold” tumor microenvironment.

Nevertheless, pulmonary NUT carcinoma exhibits heterogeneous responses to immunotherapy. Chen et al. showed that despite the poor prognosis, some cases may achieve significant responses, and even pathologic complete remission has been reported in an individual case (38).

Li et al. reviewed the clinicopathological features of all primary pulmonary NUT carcinoma patients who received immunotherapy, including initial misdiagnosis, disease stage, immunohistochemical markers related to tumor neovascularization, and biomarkers associated with immunotherapy (such as PD-L1 and TMB) (47).

A systematic review and meta-analysis (ASCO 2025 abstract) included 39 reports with 56 patients treated with pembrolizumab. The median OS was 7.3 months (95% CI: 5.2–8.8) and the median PFS was 4.6 months (95% CI: 3.1–5.7). Among three patients receiving pembrolizumab monotherapy as second-line or later treatment, the median PFS was 2.9 months. This represents the most robust evidence on immunotherapy efficacy to date and underscores the continuing challenge of treating this rare malignancy (48, 49).

One case report showed that chemotherapy combined with immunotherapy could extend OS to 15 months. Overall, however, due to the aggressive behavior of the tumor, the efficacy of chemotherapy and immunotherapy in metastatic cases remains limited (38).

Importantly, a case of primary pulmonary NUT carcinoma with WHSC1L1::NUTM1 fusion showing PD-L1 TPS of 70% was reported to respond to nivolumab for approximately 6 months. This case demonstrates that some NUT carcinoma tumors can be PD-L1-high and respond to immune checkpoint inhibitors (12). Adding checkpoint inhibitors to first-line chemotherapy for NUT carcinoma patients may also play a critical role. Therefore, the efficacy of immunotherapy should be interpreted with careful consideration of PD-L1 heterogeneity.

### 6.5 Targeted therapy

BET inhibitors (bromodomain and extra-terminal motif inhibitors) represent the most promising targeted therapy direction. BETi antagonizes the aberrant histone acetylation induced by the BRD4-NUT fusion protein, thereby suppressing tumor growth (50).

NHWD-870, a novel oral BET inhibitor independently developed in China, has made significant progress (51): It has received Breakthrough Therapy Designation from China’s National Medical Products Administration (NMPA) for advanced thoracic NUT carcinoma patients who have failed prior chemotherapy. According to a company press release and clinical trial registry data (NCT06527300), the Phase II study of NHWD-870 reported an objective response rate of 45% and a median overall survival of 9.33 months in patients with advanced thoracic NUT carcinoma. As of 27 December 2025, a total of 40 evaluable advanced NUT carcinoma patients had been enrolled. Complete response case: A 36-year-old patient with stage IIIB primary pulmonary NUT carcinoma achieved remarkable clinical efficacy with NHWD-870 monotherapy (52). Imaging showed partial response after only 1 month of treatment, and complete response was achieved within 5 months. The patient began NHWD-870 monotherapy in January 2024, rapidly achieved significant efficacy and pathological complete response, and underwent radical right lung tumor resection in June of the same year; postoperative pathology confirmed no residual cancer cells in the primary tumor or in 16 lymph nodes.

ZEN-3694 is another important BET inhibitor that should be noted: In July 2025, ZEN-3694 received FDA Fast-Track Designation (in combination with abemaciclib) for metastatic/unresectable NUT carcinoma after ≥1 prior chemotherapy line (53). In October 2025, ZEN-3694 received FDA Orphan Drug Designation for the treatment of NUT carcinoma (54). The drug is currently being evaluated in two active clinical trials: NCT05372640 (ZEN-3694 in combination with abemaciclib) and NCT05019716 (ZEN-3694 in combination with cisplatin and etoposide) (55, 56). As of April 2026, NCT05372640 had 24/40 participants enrolled. HDAC inhibitors (histone deacetylase inhibitors) have also been reported to have some therapeutic effect in NUT carcinoma. Multiple clinical trials of BETi and HDACi are currently ongoing (57).

## 7 Prognosis

Primary pulmonary NUT carcinoma has an extremely poor prognosis and is one of the lung cancer subtypes with the worst outcomes (Table 3).

**TABLE 3:** Survival estimates vary across studies.

Study	Sample size	Median OS	Mean OS
(14)	55	4.4 months	6.21 months
German cohort (39)	35	7.5 months	—
National retrospective (23)	—	8 months (95% CI: 4.09–11.91)	—

### 7.1 Survival data

Zhao et al.'s systematic review reported a median OS of 5–9 months. Primary pulmonary NUT carcinoma has a worse prognosis than NUT carcinoma at other sites.

### 7.2 Prognostic factors

- Metastasis: presence of metastasis is a risk factor for poor prognosis (HR = 2.55, 95% CI: 0.974–6.677, P = 0.057) (23).
- Age: older age at diagnosis is associated with lower cumulative survival (23).
- Sex: male sex is associated with shorter survival (23).
- Pleural effusion: presence of pleural effusion indicates shorter survival (21).
- Treatment modality: patients receiving chemoradiotherapy or surgery combined with adjuvant therapy have relatively better outcomes (14, 38).

### 7.3 Possibility of long-term survival

Despite the overall dismal prognosis, a few long-term survivors have been reported. The BMC Cancer case series documented patients surviving >12 months and >36 months (40). One patient with pulmonary NUT carcinoma developed late recurrence 14 years after initial surgery, suggesting that a minority of cases may exhibit relatively indolent biological behavior (58). These rare cases underscore the importance of aggressive treatment and long-term follow-up.

## 8 Summary and future perspectives

Primary pulmonary NUT carcinoma is a rare and highly aggressive malignancy driven by NUTM1 gene rearrangements. The disease predominantly affects young-to-middle-aged male non-smokers, with cough and dyspnea as the main symptoms. Imaging typically reveals a large lobulated pulmonary mass with lymph node and distant metastases. Pathological diagnosis relies on NUT immunohistochemistry and molecular testing; however, the morphological diversity and nonspecificity often lead to misdiagnosis.

The key to diagnosis lies in “when to think of it” rather than “how to diagnose it.” Clinicians should routinely include NUT carcinoma in the differential diagnosis of undifferentiated or poorly differentiated thoracic carcinomas, especially in young non-smoking patients. Luo et al. have suggested reclassifying NUT carcinoma as a subtype of pulmonary squamous cell carcinoma, which may help raise clinical awareness and accelerate the development of effective treatments.

Currently, there is no standardized treatment protocol. The recently published international guidelines provide the first consensus recommendations for diagnosis and treatment. Concurrent or sequential chemoradiotherapy is the most effective approach to prolong survival. BET inhibitors, particularly NHWD-870 and ZEN-3694, show breakthrough therapeutic potential. NHWD-870 phase II data demonstrate a 45% objective response rate and a median OS of 9.33 months. ZEN-3694 has received FDA Fast-Track and Orphan Drug Designations and is being evaluated in active clinical trials. Immunotherapy has limited overall efficacy due to generally low PD-L1 expression. Nevertheless, individual cases—particularly those with PD-L1-high tumors—may still achieve significant responses, underscoring the need for careful interpretation of immunotherapy efficacy in the context of PD-L1 heterogeneity.

Given the rarity and high lethality of this disease, increasing clinical awareness, early recognition, and inclusion in differential diagnosis are of paramount importance. Clinicians should encourage patients to actively participate in appropriate clinical trials. Future research should focus on elucidating the pathogenesis of NUT carcinoma, optimizing BET inhibitor strategies, exploring combination therapies (such as BETi with immunotherapy or chemotherapy), and developing new targeted agents for non-BRD4 fusions.
